# THE AMERICAN COLLEGE OF SURGEONS-NATIONAL SURGICAL QUALITY IMPROVEMENT PROGRAM CALCULATOR AND SURGICAL APGAR AS PREDICTORS OF POST-CHOLECYSTECTOMY COMPLICATIONS

**DOI:** 10.1590/0102-6720202400068e1862

**Published:** 2025-01-20

**Authors:** Diana Tejeda-Herrera, Jose Caballero-Alvarado, Carlos Zavaleta-Corvera

**Affiliations:** 1 Antenor Orrego Private University, School of Medicine, Trujillo, La Libertad, Peru.

**Keywords:** Cholecystectomy, Postoperative Complications, Bile Duct Diseases, Injury Severity Score, Bile, Colecistectomia, Complicações Pós-Operatórias, Doenças dos Dutos Biliares, Escala de Gravidade do Ferimento, Bile

## Abstract

**BACKGROUND:**

Laparoscopic cholecystectomy is considered safe; however, it is not free from complications, such as bile duct injuries, bleeding, and infection of the surgical site.

**AIMS:**

The aim of this study was to determine the effectiveness of two prediction tools, the American College of Surgeons-National Surgical Quality Improvement Program (ACS-NSQIP) calculator and the surgical Apgar, in predicting post-cholecystectomy complications.

**METHODS:**

A cross-sectional, analytical, and comparative study was conducted on patients over 18 years old diagnosed with acute cholecystitis who underwent open or laparoscopic cholecystectomy at the Regional Teaching Hospital of Trujillo between 2015 and 2019. A chi-square test was used for bivariate analysis, and the receiver operating characteristic (ROC) curve analysis was employed to determine the discriminative capacity of the ACS-NSQIP and surgical Apgar calculators in predicting severe complications.

**RESULTS:**

A total of 227 patients were included in the study. The analysis revealed that the mean age of patients who experienced severe complications was 75.32±4.58 years. Additionally, 52.6% of these patients were male. Regarding the prediction analysis based on the ROC curve, the ACS-NSQIP calculator showed an area under the curve of 0.895 (95%CI 0.819–0.971; p=0.01), whereas the surgical Apgar calculator showed an area under the curve of 0.611 (95%CI 0.488–0.735; p=0.11).

**CONCLUSIONS:**

The obtained results indicate that the ACS-NSQIP calculator is effective in predicting severe complications in patients undergoing cholecystectomy due to acute cholecystitis. These findings may have important implications for clinical practice and medical decision-making, focusing on the appropriate use of prediction tools to improve outcomes in this type of surgical procedure.

## RESUMO

**RACIONAL:**

A colecistectomia laparoscópica é considerada segura, porém não está isenta de complicações, como lesões de vias biliares, sangramento e infecção do sítio cirúrgico.

**OBJETIVOS:**

Determinar a eficácia de duas ferramentas de predição, a calculadora ACS-NSQIP e o Apgar cirúrgico, na predição de complicações pós-colecistectomia.

**MÉTODOS:**

Foi realizado um estudo transversal, analítico e comparativo em pacientes maiores de 18 anos com diagnóstico de colecistite aguda submetidos à colecistectomia aberta ou laparoscópica no Hospital Universitário Regional de Trujillo entre 2015 e 2019. O teste qui-quadrado foi utilizado para bivariado. análise, e a análise da curva ROC foi empregada para determinar a capacidade discriminativa do ACS-NSQIP e das calculadoras cirúrgicas de Apgar em predizer complicações graves.

**RESULTADOS:**

Um total de 227 pacientes foram incluídos no estudo. A análise revelou que a média de idade dos pacientes que apresentaram complicações graves foi de 75,32±14,58 anos. Além disso, 52,6% desses pacientes eram do sexo masculino. Em relação à análise de predição baseada na curva ROC, a calculadora ACS-NSQIP apresentou área sob a curva de 0,895 (IC95% 0,819–0,971; p=0,01), enquanto a calculadora cirúrgica de Apgar apresentou área sob a curva de 0,611 (IC95% 0,488–0,735; p=0,11).

**CONCLUSÕES:**

Os resultados obtidos indicam que a calculadora ACS-NSQIP é eficaz na previsão de complicações graves em pacientes submetidos à colecistectomia por colecistite aguda. Esses achados podem ter implicações importantes para a prática clínica e para a tomada de decisão médica, com foco no uso adequado de ferramentas de predição para melhorar os resultados neste tipo de procedimento cirúrgico.

## INTRODUCTION

Acute cholecystitis is defined as inflammation of the gallbladder, with gallstones having the most common etiology^1,19,21^. According to the Tokyo 2018 guidelines, this condition is classified into three grades: mild (Grade I) for healthy patients without organ dysfunction or significant inflammatory signs, moderate (Grade II) characterized by a high content of leukocytes (>18,000/mm^3^), a palpable mass in the upper right part of the abdomen, symptoms that last >72 h, and signs of local, severe inflammation (Grade III) when there is evidence of organic dysfunction^20,28^. Surgical treatment for low-risk patients involves an early cholecystectomy, whereas, for high-risk patients, drainage is performed followed by cholecystectomy once there is clinical improvement^1,7^.

Cholecystectomy, surgical removal of the gallbladder, can be performed through open or laparoscopic surgery, the latter being the standard technique^4,27^. Although laparoscopic cholecystectomy is considered safe, it is not free from complications, such as bile duct injuries, bleeding, and infection of the surgical site^4,10,15,27^.

Moderate and severe levels of acute cholecystitis are associated with a higher incidence of postoperative complications^26^, including serious complications such as pneumonia, cardiac arrest, and sepsis^9^. Therefore, it is preferable to carry out a preoperative assessment of the risk using different systems of scoring^2,11^, like the ACS-NSQIP calculator (American College of Surgeons National Surgical Quality Improvement Program calculator)^24^.

This system provides an analysis of postoperative complications in 30 days and assistance in one of the surgical decisions^13,25^. Considers 20 risk factors, offering individualized complication rates and intermediate days of hospitalization^17^. Another scoring system developed by Gawande et al.^8^, known as the surgical Apgar (AQ), eliminates postoperative complications in 30 days but is calculated after surgery. The AQ compares three intraoperative variables: the lowest heart rate, the lowest mean arterial pressure, and the estimated blood loss^23^. The results are presented as a total score, the percentage of mortality, and the rate of serious complications.

The use of scoring systems for preoperative risk assessment is crucial in patients with acute cholecystitis. It allows for better surgical planning and reduces postoperative complications^3,6^. Although laparoscopic cholecystectomy is widely considered safe, it still carries significant risks, such as bile duct injuries, bleeding, and surgical site infections^5,12^. Preoperative evaluation using the ACS-NSQIP system, which includes twenty risk factors, provides a detailed analysis of potential complications within the first 30 days and offers personalized guidance for surgical decision-making^22^. This comprehensive approach is particularly useful in high-risk procedures, where outcomes can be improved through appropriate risk stratification and the implementation of additional preventive measures.

This study aims to determine the effectiveness of the ACS-NSQIP and AQ calculators as tools for predicting complications after cholecystectomy.

## METHODS

It was carried out using a transversal, analytical, and comparative design, considering the database of the general surgery area of the Regional Teaching Hospital of Trujillo during the period 2015–2019. It included 227 patients over 18 years old diagnosed with acute cholecystitis, classified according to its severity. They underwent open or laparoscopic cholecystectomy, either electively or as an emergency. Relevant data are obtained from clinical histories, which provide information on weight and height to calculate body mass index (BMI), personal history, and the American Society of Anesthesiologists (ASA) classification to evaluate surgical risk and post-surgical complications. Anesthesia records were also compiled to obtain data on the estimated blood loss, the lowest average arterial pressure, and the lowest heart rate during the surgical intervention. Any patient who has undergone surgery for multiple different diagnoses, such as acute cholecystitis, pregnant women, and children, is excluded.

The selection of clinical histories was carried out using a simple random sample, and all information was collected and organized using Microsoft Excel. The data obtained is entered into the web links of each calculator to estimate the prediction of complications. The ACS-NSQIP calculator (https://riskcalculator.facs.org) considers the surgery to be performed in addition to 20 preoperative factors for each patient to estimate in detail the risk of complications. However, the AQ (https://www.mdcalc.com/calc/1826/surgical-apgar-score-sas-postoperative-risk) only considers three intraoperative variables and does not allow specifying the surgery to be performed. Both calculators predict the presence of mortality and complications within 30 days after surgery, with serious complications being considered the presence of pneumonia, cardiac arrest, myocardial infarction, renal failure, blood transfusion greater than four globular packets, coma, venous thrombosis deep, pulmonary embolism, cerebrovascular accident, surgical site infection in deep planes and organ/space, urinary tract infection, sepsis, septic shock, rupture of the wound, intubation, use of mechanical ventilator, and re-entry to the lung during the first 30 days post-surgical.

The statistical analysis was carried out using the Statistical Package for the Social Sciences version 26 program. For the quantitative variables, frequencies and percentages were calculated, while, for the quantitative variables, the mean and standard deviation were determined. In the bivariate analysis, the chi-squared test was used, and in addition, the receiver operating characteristic curve analysis was used to evaluate the discriminative capacity of the calculators in the prediction of complications.

The corresponding permission to carry out the investigation was obtained through resolution N°0161-2023 of the bioethics committee of the Universidad Privada Antenor Orrego, considering at all times the confidentiality of the information of the participants.

## RESULTS

A total of 227 patients undergoing cholecystectomy were evaluated, of which 19 patients presented serious complications. Table 1 presents the sociodemographic and clinical characteristics of patients undergoing cholecystectomy for acute cholecystitis, depending on the presence or absence of serious complications. Among patients with serious complications, the average age was 75.32 years, whereas in those without serious complications, it was 59.34 years. There will be no significant differences in BMI between both groups. The majority of patients with severe complications were diagnosed with Grade II and Grade III acute lithiasic cholecystitis, while the majority of patients with severe complications were diagnosed with Grade I acute lithiasic cholecystitis. It was observed that the presence of other associated diagnoses, capacity partially independent functional function, emergency surgery, sepsis, septic shock, disseminated cancer, arterial hypertension, chronic obstructive pulmonary disease (COPD), and dialysis prior to surgery were significantly associated with the appearance of serious complications (p<0.05).

**Table 1:** Sociodemographic and clinical characteristics of patients undergoing cholecystectomy for acute cholecystitis according to severe complications.

Characteristics		Severe complications		p-value
		Yes (n=19)	No (n=208)	
Age (years)		75.32 (14.58)	59.34 (18.14)	0.35
Sex (%)				
	Male	10 (52.6)	66 (31.7)	0.07
	Female	9 (47.4)	142 (68.3)	
BMI (kg/m^2^)		25.73 (3.48)	25.46 (3.84)	0.67
Diagnoses (%)				
	Acute stone cholecystitis II	8 (42.1)	99 (47.6)	0.01
	Acute stone cholecystitis III	8 (42.1)	2 (1.0)	
	Acute stone cholecystitis I	3 (15.8)	107 (51.4)	
Other diagnoses (%)				
	No	12 (63.2)	178 (85.6)	0.01
	Forks	7 (36.8)	30 (14.4)	
Functional capacity (%)				
	Independent	14 (73.7)	201 (96.6)	**0.01**
	Partially independent	5 (26.3)	7 (3.4)	
Emergency (%)				
	Emergency surgery	18 (94.7)	170 (81.7)	0.15
	Elective surgery	1 (5.3)	38 (18.3)	
Corticosteroids use (%)				
	No	19 (100.0)	207 (99.5)	0.76
	Forks	0 (0.0)	1 (0.5)	
Ascites (%)				
	No	19 (10.0)	207 (99.5)	0.76
	Forks	0 (0.0)	1 (0.5)	
Systemic inflammatory response (%)				
	No	11 (57.9)	206 (99.0)	0.01
	Sepsis	4 (21.1)	2 (1.0)	
	Septic shock	4 (21.1)	0 (0.0)	
Dependence of mechanical ventilator (%)				
	No	19 (100.0)	208 (100.0)	1.00
	Forks	0 (0.0)	0 (0.0)	
Metastasis (%)				
	No	18 (94.7)	207 (99.5)	0.03
	Forks	1 (5.3)	1 (0.5)	
Diabetes mellitus 2 (%)				
	No	16 (84.2)	189 (90.9)	0.10
	Oral hypoglycemic medication	2 (10.5)	18 (8.7)	
	Insulin	1 (5.3)	1 (0.5)	
Hypertension (%)				
	No	11 (57.9)	163 (78.4)	0.04
	Forks	8 (42.1)	45 (21.6)	
H.F. (%)				
	No	19 (100.0)	207 (99.5)	0.76
	Forks	0 (0.0)	1 (0.5)	
Dyspnea (%)				
	No	19 (100.0)	208 (100.0)	1.00
	Forks	0 (0.0)	0 (0.0)	
Tuxedo (%)				
	No	19 (100.0)	208 (100.0)	1.00
	Forks	0 (0.0)	0 (0.0)	
COPD (%)				
	No	18 (94.7)	208 (100.0)	0.01
	Forks	1 (5.3)	0 (0.0)	
Dialysis (%)				
	No	18 (94.7)	208 (100.0)	0.01
	Forks	1 (5.3)	0 (0.0)	
AKI (%)				
	No	19 (100.0)	208 (100.0)	1.00
	Forks	0 (0.0)	0 (0.0)	

BMI: body mass index; HF: heart failure; COPD: chronic pulmonary obstructive disease; AKI: acute kidney injury. Statistically significant values are denoted in bold.

Table 2 shows the preoperative and intraoperative characteristics of patients. A significant association was found between the ASA classification and the presence of severe complications (p<0.05), whereby the majority of patients with severe complications had an ASA classification of 3. Furthermore, significant differences were observed in the highest heart rate and lower mean arterial pressure was lower between both groups (p<0.05), being greater in patients with serious complications. No significant differences were found in the estimated blood loss between the groups.

**Table 2:** Preoperative and intraoperative characteristics of patients undergoing cholecystectomy for acute cholecystitis according to severe complications.

Characteristics		Severe complications		p-value
		Yes (n=19)	No (n=208)	
ASA classification (%)					
	ASA 3	10 (52.6)	19 (9.1)	0.01
	ASA 2	7 (36.8)	102 (49.0)	
	ASA 1	1 (5.3)	86 (41.3)	
	ASA 4	1 (5.3)	1 (0.5)	
Lower heart rate (bpm)		75.42 (15.20)	71.04 (9.72)	0.04
Lower mean arterial pressure (mmHg)		72.37 (14.52)	76.13 (9.64)	0.01
Estimated bleeding loss (mL)		197.37 (189.64)	183.94 (178.89)	0.43

ASA: American Society of Anesthesiologists; bpm: beats per minute; mmHg: milimeters of mercury; mL: milliliters.

Table 3 presents the postoperative characteristics of the patients. A significant association was observed between the type of cholecystectomy performed and the presence of serious complications (p<0.05). Of the total number of patients who suffered serious complications, 7.9% had undergone open cholecystectomy, while only 0.4% had undergone laparoscopic cholecystectomy. In contrast, the majority of patients without severe complications (91.6%) had undergone open (63.4%) or laparoscopic (28.2%) cholecystectomy.

**Table 3:** Postoperative characteristics of patients undergoing cholecystectomy for acute cholecystitis.

	Severe complications		Total (%)	p-value
	Forks (%)	No (%)		
Open cholecystectomy	18 (7.9)	144 (63.4)	162 (71.4)	0.02
Laparoscopic cholecystectomy	1 (0.4)	64 (28.2)	65 (28.6)	
Total	19 (8.4)	208 (91.6)	227 (100.0)	

Table 4 presents the results obtained using prediction calculators, ACS-NSQIP, and surgical Apgar. For the risk of serious complications, the ACS-NSQIP calculator classified 55.5% of patients as having a lower risk than average, while 44.5% had a risk equal to greater than average. On the other hand, the surgical Apgar calculator classified 76.7% of patients as having a risk lower than average and 23.3% as having a risk equal to greater than average. In terms of mortality risk, both calculators gave similar results, where 83.3% of patients had a lower risk compared to the average and 16.7% had a risk equal to the greater average according to the ACS-NSQIP calculator, while the surgical Apgar calculator It also showed that 76.7% had a lower risk than the average and 23.3% had a risk equal to the greater risk at the average.

**Table 4:** Predictive calculator.

ACS-NSQIP calculator			n	%
Risk of severe complications (mean: 7.20±5.06)				
	Lower at the average risk		126	55.5
	Equal or greater at the average risk		101	44.5
Risk of mortality (mean: 0.76±1.97)				
	Lower at the average risk		189	83.3
	Equal or greater at the average risk		38	16.7
Surgical Apgar calculator				
	Risk of severe complications (mean: 8.74±7.68)			
		Lower at the average risk	174	76.7
		Equal or greater at the average risk	53	23.3
	Risk of mortality (mean: 1.73±2.14)			
		Lower at the average risk	174	76.7
		Equal or greater at the average risk	53	23.3

ACS-NSQIP: American College of Surgeons-National Surgical Quality Improvement Program.

**Figure 1:**
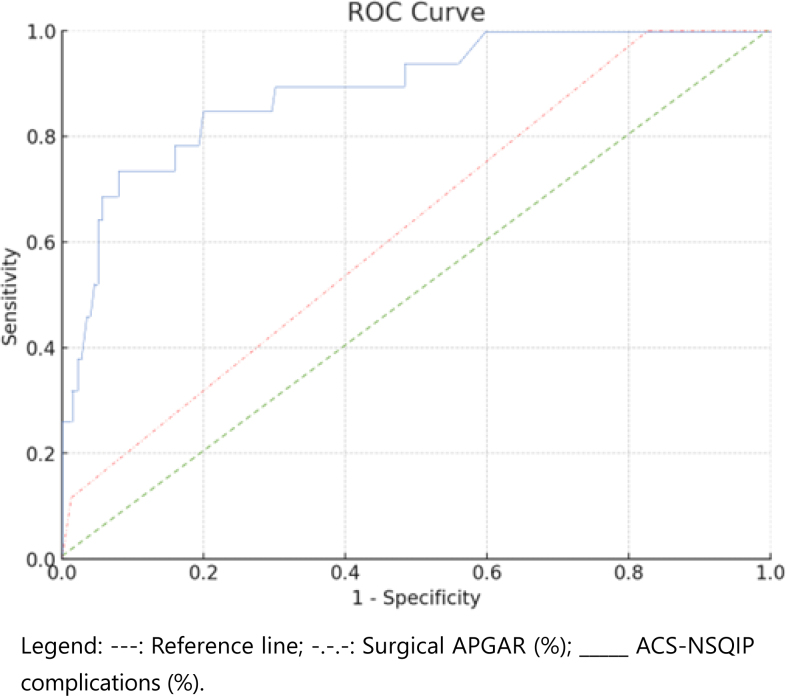
Analysis of serious complication prediction calculators.

Figure 1 shows the analysis of serious complication prediction calculators, ACS-NSQIP and surgical Apgar, in patients undergoing cholecystectomy for acute cholecystitis. The area under the curve was evaluated as a measure of the discriminative capacity of each calculator. The results reveal that the ACS-NSQIP calculator obtained an area below the curve of 0.895 (95%CI 0.819–0.971), which indicates a high capacity to predict serious complications. On the other hand, the surgical Apgar calculator showed an area below the curve of 0.611 (95%CI 0.488–0.735), which indicates a limited ability to predict serious complications.

## DISCUSSION

The results obtained in this study show that the advanced age of patients is significantly associated with a greater risk of serious complications after cholecystectomy. This highlights the importance of taking precautions and taking preventive measures in older patients, as they may be more exposed to complications, as reported by D’Acapito et al.^6^, and Kamarajah et al.^14^, Furthermore, we can find some information for additional diagnoses, such as disseminated cancer, arterial hypertension, COPD, and the need for dialysis prior to surgery, are also significantly associated with a greater risk of serious complications, which suggests that these comorbidity factors may influence the postoperative prognosis^3,16^.

The ASA classification also proved to be an important factor in the prediction of serious complications, where patients with an ASA classification of 3 had the highest incidence of complications. This classification is based on the assessment of the patient's physical state before surgery and is commonly used to predict the surgical risk. The results suggest that patients with an ASA classification of 3 may require more careful evaluation and additional preventive measures before surgery to reduce the risk of complications, which differs from that evidenced by Mastalerz et al.^18^, who mention that the ASA classification alone is a predictor of morbidity and suggest including intraoperative evaluation to predict post-surgery complications.

Regarding prediction calculators, the ACS-NSQIP demonstrated a high capacity to predict serious complications, classifying patients at low or high risk with high precision. On the other hand, the surgical Apgar calculator showed a limited ability to predict serious complications.

These findings are consistent with other studies that have supported the effectiveness of the ACS-NSQIP calculator in several surgical procedures to predict postoperative complications^2,12,22^. However, it is important to mention that no prediction calculator is infallible, and using several tools together can provide a more complete and accurate vision of the postoperative risk.

Despite promising results, it is necessary to continue investigating and evaluating other prediction calculators in broader populations and in different surgical specialties to improve their predictive capacity and adapt them to the specific situations of each patient. Furthermore, it must be considered that postoperative complications on the ground depend on clinical and demographic factors, as well as on the skill and experience of the surgical team, the quality of postoperative care, and other institutional factors. Therefore, a comprehensive and multidisciplinary approach is essential to improving surgical results and patient safety.

Future investigations could focus on analyzing specific risk factors associated with different postoperative complications in cholecystectomy for acute cholecystitis, to identify specific factors for each type of complication, and to design more targeted and effective interventions and prevention strategies. On the other hand, other prediction calculators could be validated that will allow determining which is most appropriate for this population of patients and could help identify new relevant risk factors. Another line of interesting research could be the development and evaluation of preventive interventions aimed at reducing the incidence of serious complications in patients undergoing cholecystectomy for acute cholecystitis.

## CONCLUSIONS

The study provides valuable information on factors associated with serious complications in patients undergoing cholecystectomy for acute cholecystitis. In advanced age, certain additional diagnoses and a higher ASA classification were identified as significant risk factors. Furthermore, the usefulness of the ACS-NSQIP calculator to predict serious complications stands out, which can help clinicians make informed decisions and improve the quality of surgical care. However, it is recommended to continue investigating and comparing other predictive tools to continue advancing toward improving safety and results in surgery.

Central Message Although laparoscopic cholecystectomy for acute cholecystitis is considered safe, it is not free from complications, such as bile duct injuries, bleeding, and infection of the surgical site, including serious complications such as pneumonia, cardiac arrest, and sepsis. Therefore, it is preferable to carry out a preoperative assessment of the risk using different systems of scoring, like the American College of Surgeons-National Surgical Quality Improvement Program calculator. Another scoring system option is known as the surgical Apgar (AQ).

Perspectives The study provides valuable information on factors associated with serious complications in patients undergoing cholecystectomy for acute cholecystitis. The advanced age, certain additional diagnoses, and a higher American Society of Anesthesiologists classification were identified as significant risk factors. Furthermore, the usefulness of the American College of Surgeons-National Surgical Quality Improvement Program calculator to predict serious complications stands out, which can help clinicians make informed decisions and improve the quality of surgical care.
